# Protein-protein interaction analysis of distinct molecular pathways in two subtypes of colorectal carcinoma

**DOI:** 10.3892/mmr.2014.2585

**Published:** 2014-09-22

**Authors:** HANZHANG CHEN, YUNZHEN FANG, HAILONG ZHU, SHUAI LI, TAO WANG, PAN GU, XIA FANG, YUNJIN WU, JUN LIANG, YU ZENG, LONG ZHANG, WEIZHE QIU, LANJING ZHANG, XIANGHUA YI

**Affiliations:** 1 Department of Pathology, Central Hospital of Shanghai Zhabei District, Shanghai 200070, P.R. China; 2 The Operating Room, Central Hospital of Shanghai Zhabei District, Shanghai 200070, P.R. China; 3 Department of Pathology, Tongji Hospital, Tongji University School of Medicine, Shanghai Tongji Hospital, Shanghai 200065, P.R. China; 4 Urology Surgery, The First People’s Hospital of Jingzhou, Hubei 434000, P.R. China; 5 Hematology Department, Tongji Hospital, Tongji University School of Medicine, Shanghai Tongji Hospital, Shanghai 200065, P.R. China; 6 Department of Pathology, University Medical Center of Princeton, Plainsboro, NJ 08536, USA; 7 Department of Pathology, Rutgers Robert Wood Johnson Medical School and Rutgers Cancer Institute of New Jersey, New Brunswick, NJ 08903-2601, USA

**Keywords:** microarray, serrated colorectal carcinoma, conventional colorectal carcinoma, proteasome pathway, TRAF6, ATN1

## Abstract

The aim of this study was to identify the molecular events that distinguish serrated colorectal carcinoma (SCRC) from conventional colorectal carcinoma (CCRC) through differential gene expression, pathway and protein-protein interaction (PPI) network analysis. The GSE4045 and GSE8671 microarray datasets were downloaded from the Gene Expression Omnibus database. We identified the genes that are differentially expressed between SCRC and normal colon tissues, CCRC and healthy tissues, and between SCRC and CCRC using Student’s t-tests and Benjamini-Hochberg (BH) multiple testing corrections. The differentially expressed genes (DEGs) were then mapped to Kyoto Encyclopedia of Genes and Genomes (KEGG) pathways and their enrichment for specific pathways was investigated using the Database for Annotation, Visualization and Integrated Discovery (DAVID) tool with a significance threshold of 0.1. Analysis of the potential interactions between the protein products of 220 DEGs (between CCRC and SCRC) was performed by constructing a PPI network using data from the high performance RDF database (P<0.1). The interaction between pathways was also analyzed in CCRC based on the PPI network. Our study identified thousands of genes differentially expressed in SCRC and CCRC compared to healthy tissues. The DEGs in SCRC and CCRC were enriched in cell cycle, DNA replication, and base excision repair pathways. The proteasome pathway was significantly enriched in SCRC but not in CCRC after BH adjustment. The PPI network showed that tumour necrosis factor receptor-associated factor 6 (TRAF6) and atrophin 1 (ATN1) were the most central genes in the network, with respective degrees of node predicted at 90 and 88. In conclusion, the preoteasome pathway was shown to be specifically enriched in SCRC. Furthermore, *TRAF6* and *ATN1* may be promising biomarkers for the distinction between serrated and conventional CRC.

## Introduction

Colorectal carcinoma (CRC) is the third most commonly diagnosed cancer (1) and has been reported to occur through different pathways; approximately one third of CRC cases arise along the serrated pathway developing from sessile serrated adenoma (2). The term ‘serrated adenoma’ was first proposed in 1990 by Longacre and Fenoglio-Preiser (3). Morphological features can allow a preliminary distinction of forms of colorectal cancer. The morphological characteristics of serrated CRC (SCRC) include a more mature epithelium than that of conventional CRC (CCRC) (4). However, morphological features are not sufficient for diagnosis of this tumor type.

Serrated adenoma is related to genetic alterations, including DNA methylation, DNA microsatellite instability, *K-ras* mutation and loss of chromosome 1p (5). Sessile serrated adenomas can induce carcinomas with extensive CpG island promoter methylation, which can be either microsatellite-instable high or microsatellite stable (2). The gene mutated in colorectal cancer was shown to selectively repress β-catenin-dependent transcription, and was thus proposed to constitute a tumor suppressor gene in the SCRC pathway (6). The *c-MYC* oncogene was shown to be activated by the MAPK/ERK1/2 pathway via the genes *K-ras* and *BRAF* and by Wnt signalling in the serrated pathway (7). A mutation in *BRAF* was frequently observed in serrated adenoma tissues associated with DNA methylation (8). The genes encoding ephrin receptor B2, hypoxia-inducible factor 1-α, and patched were reported as important for the genesis of SCRC (4). The expression of genes *p53*, *APC* and *CRAC* was found altered in SCRC as well as in CCRC (9). However, the mechanism(s) underlying the pathogenesis of SCRC are still not well elucidated.

In this study, we identified differentially expressed genes (DEGs) between SCRC and CCRC and between each of these subtypes of CRC and healthy tissues. Then, DEGs were analyzed for their enrichment in molecular pathways, and a protein-protein interaction (PPI) network was constructed to identify the potential interactions between the genes’ products. Overall, this study provided information that may be useful for the clinical classification of CRC.

## Materials and methods

### Microarray data

The transcriptional profile data of SCRC samples, CCRC samples and healthy mucosa samples were downloaded from the Gene Expression Omnibus (GEO; http://www.ncbi.nlm.nih.gov/geo/). The data were derived from the accession numbers GSE4045 (4) and GSE8671 (10). GSE4045 comprises7 SCRC and 30 CCRC samples. CRC samples with serrated histology were from two population-based Finnish collections. The analysis of microarray data was performed on a GPL96 (HG-U133A) Affymetrix Human Genome U133A array. The GSE8671 dataset comprises 32 CCRC samples and 32 healthy mucosa samples, which were obtained from patients during colonoscopy. The analysis of microarray data was performed on a GPL570 (HG-U133-Plus_2) Affymetrix Human Genome U133 Plus 2.0 array. The raw data (CEL files) from both datasets were downloaded.

### Normalization of microarray data and identification of DEGs

First, data from each dataset were separately normalized. The two sets of data were preprocessed using background correction and robust multi-array analysis (RMA) normalization (11) with the Affy package (12). From the chip expression profile data, we removed the probes that corresponded to multiple Entrez Gene IDs and retained the median of different probes representing the same Entrez Gene ID. We finally obtained expression profile data for 12,779 genes from 37 samples of the GSE4045 dataset and for 20,539 genes from 64 samples of the GSE8671 dataset. Second, the datasets derived from the different platforms were normalized between them. It has been reported that batch effects are critical in high-throughput data (13). Thus, cross-platform normalization (XPN) was used (14). We selected the 12,779 common genes between the two datasets and their expression data were normalized with the XPN method using the CONOR package (15). These data were used for further analyses. The DEGs were identified from the normalized final dataset using Student’s t-tests (P<0.05), followed by Benjamini-Hochberg (BH) multiple testing correction (16). A BH P-value <0.05 was set as the threshold.

### Construction of a cancer global network

In order to explore expression patterns in the two CRC subtypes, we constructed a cancer global network. First, we downloaded the following 14 cancer-related pathways from the Kyoto Encyclopedia of Genes and Genomes (KEGG) pathway database (17): colorectal cancer, pancreatic cancer, glioma, thyroid cancer, acute myeloid leukemia, chronic myeloid leukemia, basal cell carcinoma, melanoma, renal cell carcinoma, bladder cancer, prostate cancer, endometrial cancer, small cell lung cancer and non-small cell lung cancer. Second, we screened the DEGs which were enriched in the 14 cancer pathways and then a network of these DEGs was constructed.

### KEGG pathway enrichment analysis

The Database for Annotation, Visualization and Integrated Discovery (DAVID) online enrichment tool (18,19) was adopted for pathway enrichment analysis, and the EASE score (a modified Fisher exact test) was used to identify significantly enriched KEGG pathways with a threshold value of 0.1. BH adjustment was performed on the raw P-values obtained from the pathway enrichment analysis.

### PPI network construction

We constructed a PPI network for 149 DEGs selected from the GSE4045 dataset (P-value after BH adjustment <0.1) using the high performance RDF database (HPRD) (20). In the PPI network, a hub was defined as the node which has the most number of interactions with other nodes. PPI was visualized using the Cytoscape software (21).

### Construction of an interactions network of pathways involved in SCRC and CCRC based on the PPI network

Significantly enriched pathways of DEGS were used to construct a pathway interaction network. We used the cumulative probability of the hypergeometric distribution model to identify whether any two pathways included a significant number of interactions (P<0.05). The cumulative probability formula was as follows:

$$p=1-\sum_{k=0}^{m-1}\frac{\left(\begin{array}{c} M\\ k \end{array}\right)\;\left(\begin{array}{c} N-M\\ n-k \end{array}\right)}{\left(\begin{array}{c} N\\ n \end{array}\right)}$$

where *N* represents the number of protein interactions in which all DEGs are involved; *M* represents the number of protein interactions which are related to genes in pathway 1 (there was at least one DEG in the interaction); *n* represents the number of protein interactions that are related to genes in pathway 2 (there was at least one DEG in the interaction); *k* represents the number of interactions between pathway 1 and 2 (there was at least one DEG in the interaction). If the cumulative probability was less than 0.1, two pathways were defined as interacting. We defined the degree as the number of interactions which one node had with other nodes.

## Results

### Identification of DEGs in SCRC and CCRC

In this study, we set two different significance thresholds to identify DEGs, listed in Table I. When P<0.05, the proportion of upregulated genes was higher in CCRC (62.6%, 1,713/2,736) compared to SCRC (36.9%, 1,012/2,736). Using a Fisher exact test, we found that there was a significant difference in the proportion of up- and downregulated genes between these two cancer subtypes (P=2.596e-08). Then, we adjusted the P-value using BH multiple testing correction.

### Global cancer network

**Figure 1:**
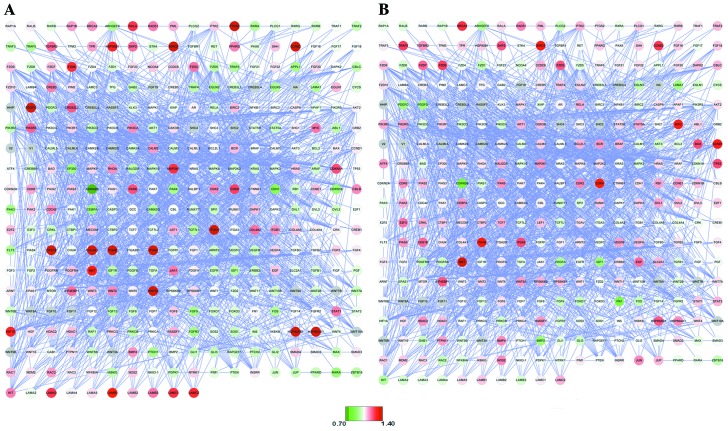
Global cancer network in (A) serrated colorectal carcinoma and (B) conventional colorectal carcinoma. Red circles represent upregulated genes and green circles downregulated genes.

We constructed a network of 341 genes and 1,569 relation after identifying the DEGs which were enriched in the 14 cancer pathways (Fig. 1). Then, information on the expression profile of these genes was retrieved for DEGs in the two subtypes of CRC as shown in Fig. 1. In SCRC, there were 130 upregulated genes (48 genes of these appear downregulated in CCRC) and 184 downregulated genes (80 of these appear upregulated in CCRC). In CCRC, there were 162 upregulated and 152 downregulated genes. A total of 182 genes showed expression changes in the same direction in the two networks, comprising 100 downregulated and 82 upregulated genes.

After a Fisher exact test, there were 26 upregulated genes in SCRC and 25 upregulated genes in CCRC when P<0.05. In addition, there were 13 genes which were both upregulated between the two CRC subtypes with P<0.05. There were 12 genes with a fold-change (FC) >1.4 and one gene with FC<0.7 in SCRC, and 1 gene with FC>1.4 and no gene with FC<0.7 in CCRC.

### Identification of biological pathways related to SCRC and CCRC

In this study, 2,736 DEGs from CCRC and 2,123 DEGs from SCRC (P<0.05; Table I) were selected for pathway enrichment analysis. Table II shows the top 9 most significantly enriched pathways in CCRC, in which are involved the genes with a BH-adjusted P-value <0.05. These significantly enriched pathways can be divided into three classes: pathways related to death (such as cell cycle and p53 signaling pathway); DNA replication and repair pathways (such as DNA replication, base and nucleotide excision repair, mismatch repair, and homologous recombination); and nucleotide metabolism-related pathways (such as purine and pyrimidine metabolism).

Table III shows the top 10 most significantly enriched pathways in SCRC, in which are involved the genes with a P<0.05. These pathways can also be divided in three classes: cell growth and death pathways (cell cycle); DNA replication and repair pathways (DNA replication and base excision repair); nucleotide metabolism-related pathways (such as purine and pyrimidine metabolism).

**Figure 2:**
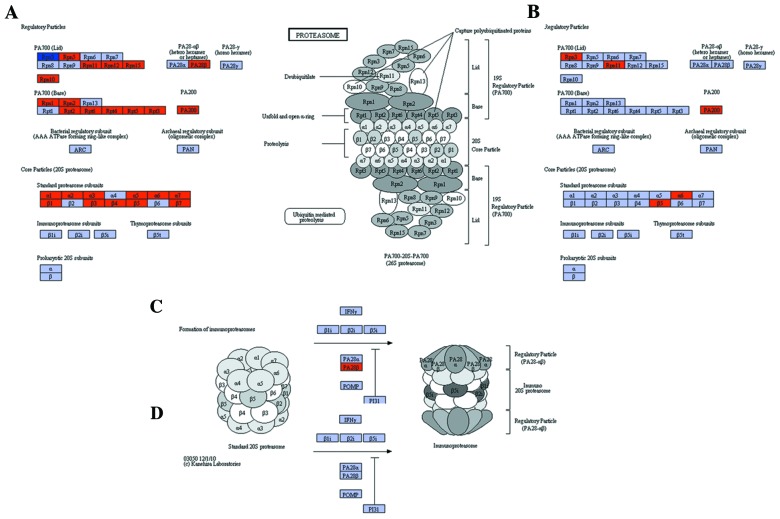
Differentially expressed genes in the proteasome pathway. Red boxes represent upregulated and dark blue boxes downregulated genes in cancer samples. Regulatory particles in (A) serrated and (B) conventional colorectal carcinoma and formation of immunoproteasomes in (C) serrated and (D) conventional colorectal carcinoma. Source, Kyoto Encyclopedia of Genes and Genomes (KEGG), *Homo sapiens* pathways.

Following BH adjustment, the proteasome pathway was the only significantly enriched pathway in SCRC (P=7.18E-0.08). The proteasome pathway included 26 DEGs in SCRC, but was not enriched in CCRC. The proteasome pathway and the related DEGs are shown in Fig. 2. Nearly all DEGs in the proteasome pathway were upregulated genes, and the number of upregulated genes in SCRC (Fig. 2A and C) was higher compared to CCRC (Fig. 2B and D). The expression pattern of the gene *Rpn3* was different between SCRC and CCRC: the gene was found to be downregulated in SCRC (Fig. 2B) and upregulated in CCRC (Fig. 2A).

### Pathway interactions model based on PPI

**Figure 3:**
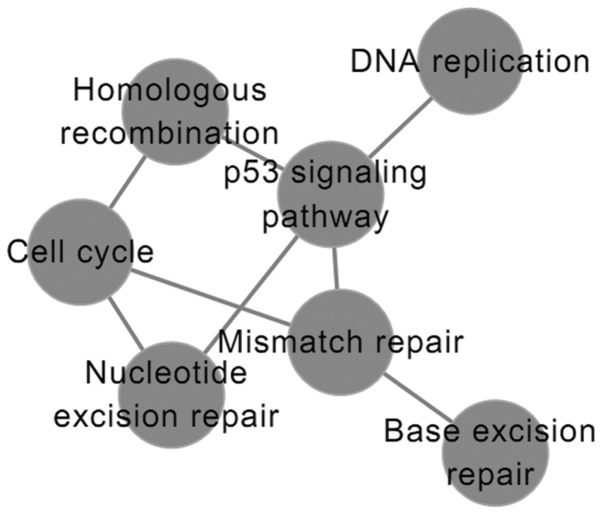
Pathway network in conventional colorectal carcinoma. Nodes represent significantly enriched pathways. Edges represent the interaction between pathways.

There was no interactions among pathways involved in SCRC (data not shown). However, there was some interaction between significantly enriched pathways involved in CCRC (Fig. 3). In the interaction network, p53 signaling, cell cycle and mismatch repair pathways showed the highest degree of nodes.

### PPI network

**Figure 4:**
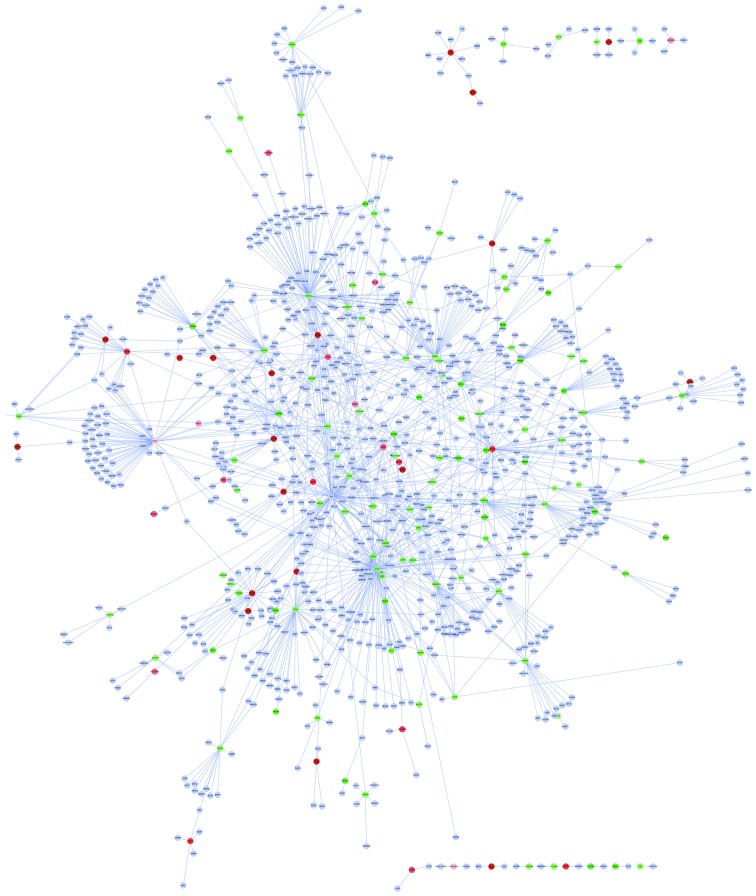
Protein-protein interaction network of the 149 differential expressed genes (DEGs). Red nodes represent upregulated and green nodes downregulated gene products (proteins). Gray nodes represent non-differentially expressed proteins interacting with DEG gene products.

We constructed a PPI network for 149 DEGs involved in a total of 1,564 protein interactions. As shown in Fig. 4, we found 5 hub DEGs, i.e., DEGS that show the highest degree of nodes: tumour necrosis factor receptor-associated factor 6 (*TRAF6*, P=0.088453), atrophin 1 (*ATN1*, P=0.055616), integrin β1 (*ITGB1*, P=0.037011), fragile X-related gene 2 (*FXR2*, P=0.075519), kinase γ (*IKBKG*, P=0.094505) with degrees of node estimated at 90, 88, 62, 53 and 45, respectively (Table IV).

## Discussion

Serrated and conventional CRC are two subtypes of CRC. In this study, we attempted to identify the similarities and differences between these subtypes. DEGs were identified, and the enriched pathways in which these DEGs are involved were analyzed. We also constructed pathway interaction networks and PPI networks to further understand the molecular mechanisms underlying the pathogenesis of these two subtypes.

Based on KEGG pathway enrichment analysis, significantly enriched pathways in SCRC and CCRC included cell cycle, DNA replication, and base excision repair. These results suggest that a number of pathways are common between SCRC and CCRC.

The proteasome pathway was the only pathway that was enriched in SCRC but not in CCRC after BH adjustment. Proteasome is a subcellular organelle that is distributed in the cytosol, nucleus, endoplasmic reticulum and lysosome of eukaryotic cells (22). Its main function is the digestion of proteins, damaged or misfolded proteins, participation in the synthesis of peptides in the immune system, and regulation of the survival (half-life) of proteins that control the cell cycle. More than 80% of the proteins are processed by the proteasome in the cells (23). Proteasome dysfunction leads to disorders in cell-cycle regulation, cell hyperplasia, and imbalances in positive and negative signals (23). Inhibition of the proteasome function can lead to cell-cycle arrest, leading to cell death (24). Targeting the proteasome has been proposed as a novel approach in cancer therapy (25). Bortzomib is a proteasome inhibitor with antitumor activity against, for example, CRC, as shown *in vitro* (25), but it was revealed to be inactive in the clinic for patients with metastatic colorectal cancer (26). Our results showed that DEGs in CCRC were not significantly enriched for the proteasome pathway, which would provide an explanation for the clinical failure of bortzomib. It was also reported that the proteasome inhibitor MG-132 inhibits growth and stimulates cell apoptosis in CRC (27). In the present study, the proteasome pathway was enriched in SCRC, but not in CCRC. Therefore, the proteasome seems to be a pathway specifically involved in SCRC. The exact involvement of this pathway in SCRC merits further investigation.

In the CCRC pathway interaction network, we observed some interaction between pathways that was not observed in the SCRC pathway interaction network. The p53, cell cycle and mismatch repair pathways showed the highest degree of node. A mutation in *p53* was previously reported to be detectable in the plasma and serum of patients with colorectal cancer or adenomas (28). Moreover, adenovirus-mediated transfer of genes of the p53 family induced cell-cycle arrest in colorectal cancer (29). It was also reported than mutations in *AXIN2* cause colorectal cancer with defective mismatch repair by activating β-catenin or TCF signaling (30). Thus, our results suggest that the interaction between pathways may be important in the pathogenesis of conventional CRC.

In the PPI network, there were five hub genes, *TRAF6*, *ATN1*, *ITGB1*, *FXR2* and *IKBKG*. *TRAF6* had the highest degree of node. *TRAF6* encodes a ubiquitin ligase. When activated, it can produce short protein chains. TRAF6 acts as a molecular switch, which allows activation of different signals. TGF with TRAF6 were reported to specifically activate the kinase TAK1 and other stress-activated kinases, leading to cell death (31). *TRAF6* was shown to be an important oncogene for RAS-mediated oncogenesis in lung cancers (32). Ubiquitination plays a critical role in the activation of the nuclear factor-κB (NF-κB) signaling pathway, which has multiple functions in regulating cell proliferation, apoptosis and immune responses (33). There is no study to date on the involvement of *TRAF6* in CRC. *ATN1* encodes a nuclear corepressor expressed in nervous tissue. It interacts with tumor suppressors to control planar polarity (34). There are only a few reports on *ATN1* and cancer (35,36). Therefore, *TRAF6* and *ATN1* may represent novel biomarkers, which are suitable for distinguishing SCRC and CCRC.

In conclusion, we found a number of significantly enriched pathways in SCRC and CCRC, which revealed certain aspects of the pathogenesis of these two subtypes of CRC. We also found that the proteasome pathway is significantly enriched only in SCRC. In addition, we identified the genes *TRAF6* and *ATN1*, which showed the highest degree of node in the constructed PPI network. These results will be helpful for the understanding of the genesis and the specific therapy of SCRC and CCRC. However, further experiments are needed to confirm our results.

## Figures and Tables

**Table I:** Identification of differentially expressed genes (DEGs), numbers obtained from application of different criteria.

	P<0.05		
	
	Total	Upregulated	Downregulated
CCRC vs. SCRC	1,819	602	1,299
CCRC vs. control	2,736	1,713	1,023
SCRC vs. control	2,123	1,012	1,121

CCRC, conventional colorectal carcinoma; SCRC, serrated colorectal carcinoma; control, healthy mucosa samples.

**Table II:** Enriched pathways for differentially expressed genes (BH P<0.05) in conventional colorectal carcinoma. Source, Kyoto Encyclopedia of Genes and Genomes (KEGG), *Homo sapiens* (hsa) identification numbers.

KEGG id.	Gene count	BH
04110: Cell cycle	61	2.53E-12
03030: DNA replication	27	3.41E-10
00230: Purine metabolism	55	5.07E-05
00240: Pyrimidine metabolism	38	2.74E-04
03410: Base excision repair	20	3.12E-04
03420: Nucleotide excision repair	21	5.45E-03
03430: Mismatch repair	14	8.23E-03
04115: p53 signaling pathway	27	1.46E-02
03440: Homologous recombination	15	2.47E-02

BH, P-value after Benjamin-Hochberg correction.

**Table III:** Enriched pathways for differentially expressed genes (P<0.05) in serrated colorectal carcinoma. Source, Kyoto Encyclopedia of Genes and Genomes (KEGG), *Homo sapiens* (hsa) identification numbers.

KEGG id.	Gene count	BH
03050: Proteasome	26	7.18E-08
04110: Cell cycle	31	3.82E-01
04512: ECM-receptor interaction	23	4.29E-01
05222: Small cell lung cancer	22	7.11E-01
05200: Pathways in cancer	64	7.68E-01
04914: Progesterone-mediated oocyte maturation	22	8.08E-01
03030: DNA replication	12	8.44E-01
00240: Pyrimidine metabolism	23	9.28E-01
03410: Base excision repair	11	9.84E-01

BH, P-value after Benjamin-Hochberg correction.

**Table IV:** Information on the hub genes in the PPI network.

Gene symbol	Degree	Dir	P-value_BH
*TRAF6*	90	Downregulated	0.088453
*ATN1*	88	Downregulated	0.055616
*ITGB1*	62	Upregulated	0.037011
*FXR2*	53	Downregulated	0.075519
*IKBKG*	45	Downregulated	0.094505

PPI, protein-protein interaction; Dir, direction BH, Benjamini-Hochberg; TRAF6, tumour necrosis factor receptor-associated factor 6; ATN1, atrophin 1; ITGB1, integrin β1; FXR2, fragile X-related gene 2; IKBKG, inhibitor of nuclear factor κ-B kinase subunit γ.
